# Targeting F2R/PAR1 with ligand decorated lipid nanocarriers for enhanced drug delivery into ovarian cancer cells

**DOI:** 10.3389/fddev.2025.1727958

**Published:** 2026-01-13

**Authors:** Riya Khetan, Weranga Rajapaksha, Bukuru D. Nturubika, Todd A. Gillam, Doug A. Brooks, Sanjay Garg, Anton Blencowe, Hugo Albrecht, Preethi Eldi

**Affiliations:** 1 Centre of Pharmaceutical Innovation, UniSA Clinical and Health Sciences, University of South Australia, Adelaide, SA, Australia; 2 UniSA Clinical and Health Sciences, University of South Australia, Adelaide, SA, Australia; 3 School of Mathematics, Statistics, Chemistry and Physics, Murdoch University, Murdoch, WA, Australia; 4 Applied Chemistry and Translational Biomaterials Group, Centre of Pharmaceutical Innovation, UniSA Clinical and Health Sciences, University of South Australia, Adelaide, SA, Australia

**Keywords:** active targeting, F2R/PAR1, ligand-decorated nanocarriers, lipid nanoparticles, ovarian cancer

## Abstract

Ovarian cancer treatment by chemotherapy is often complicated by severe systemic toxicity, highlighting the need for targeted delivery techniques that can improve drug efficacy while minimizing off-target effects. Our previous research identified the G protein-coupled receptor (GPCR), coagulation factor II thrombin receptor/protease activated receptor 1 (F2R/PAR1), as a potential therapeutic target in metastatic ovarian cancer tissues. Here we report the design of an engineered lipid nanoparticle (LNP), conjugated with a synthetic short peptide agonist that mimics the F2R-activating tethered ligand. Doxorubicin (DOX)-loaded LNPs (LNP-DOX), were physically characterized to assess the drug encapsulation efficacy, particle size, polydispersity index (PDI), zeta potential, and release kinetics. *In vitro* investigation demonstrated that the peptide-conjugated LNPs had significantly increased cellular uptake and cytotoxicity compared to their non-conjugated equivalents in an established ovarian cancer cell line. The results underscore the therapeutic potential of ligand-directed nanocarriers for targeted drug delivery into ovarian cancer cells and further validates F2R as a promising cell surface target.

## 1 Introduction

Ovarian cancer continues to be one of the most lethal gynecological cancers, primarily due to its late-stage diagnosis, rapid resistance to chemotherapy and frequent recurrence post-treatment. The survival rate for most patients diagnosed with advanced-stage disease is less than 5 years. Although advances have been made with platinum-based chemotherapy and cytoreductive surgery (Beachler et al., 2020; Narod, 2016), the highly aggressive disease phenotype remains largely refractory. There is an urgent need for improved therapeutic delivery methods that can precisely target tumor cells and be efficiently realized. Nanoparticle-based drug delivery systems have considerable potential to achieve this objective by improving drug solubility, extending vascular circulation duration, and facilitating targeted distribution to tumor sites by both passive and active processes (Dang and Guan, 2020; Khetan et al., 2022; Yao et al., 2020; Yu et al., 2020).

Lipid nanoparticles (LNPs) have emerged as promising nanocarriers for clinical translation due to well established scalable manufacture, biocompatibility, and the potential to encapsulate both hydrophobic and hydrophilic chemotherapeutic drugs (Dilliard and Siegwart, 2023; Mehta et al., 2023; Seo et al., 2023). Multiple LNP formulations, including Doxil^®^ and Myocet^®^, use liposomal encapsulation of DOX, and have demonstrated clinical efficacy and attained regulatory approval (Barenholz, 2012; Mross et al., 2004). However, most of these nanoparticle systems depend on passive accumulation via the enhanced permeability and retention (EPR) effect, which has proven to have limited effect in ovarian cancer due to the complex tumor microenvironment and formation of protein coronas on the surface of nanoparticles (Khetan et al., 2022). Ovarian cancer is known to spread within the peritoneal cavity via spheroids diffusing through ascitic fluid (Rakina et al., 2022). In this situation, the efficient distribution of passively targeted nanoparticles is reduced by compromised lymphatic outflow (Khetan et al., 2022). While intraperitoneal administration of free chemotherapeutics, such as cisplatin or carboplatin, can enhance local drug exposure, their clinical utility has been restricted due to severe off-target toxicities (Bouchard-Fortier et al., 2016). Consequently, there is an increasing emphasis on the development of actively targeted nanocarriers, which are designed to specifically recognize and bind to overexpressed cell surface receptors on tumor cells (Liu et al., 2025). These ligand-directed strategies not only enhance the selectivity and uptake of nanoparticles but can also potentially reduce non-specific toxicity and increase therapeutic efficacy.

G-protein coupled receptors (GPCRs) play crucial roles in tumor biology and have been largely underutilized as therapeutic cancer targets, especially for ovarian cancer (Feigin, 2013; Liu et al., 2016). GPCRs are highly expressed in cancer cells and are readily accessible on the cell surface, rendering them as appropriate docking sites for targeted therapeutics (Li et al., 2005). We previously used publicly available RNA sequence datasets to identify a selection of GPCRs with elevated expression in ovarian cancer patient samples (Khetan et al., 2024), and shortlisted F2R for the development of a targeted drug carrier, due to its high level of expression specifically in metastatic and chemotherapeutic resistant tumors (Khetan et al., 2025). The F2R is a thrombin-activated GPCR involved in specific oncogenic processes, such as migration, invasion, epithelial-mesenchymal transition, and chemoresistance, making this receptor a promising therapeutic target (Dong and Bao, 2024; Gao et al., 2020; Jiang et al., 2021). The increased expression of F2R in metastatic and chemotherapy-resistant ovarian cancer tissues (Khetan et al., 2025), makes this an ideal potential targeting system to treat late-stage/aggressive ovarian cancer.

Protease activated receptors (PARs) like F2R represent a distinct category of GPCRs that are triggered by irreversible proteolytic cleavage instead of just traditional ligand binding. Following cleavage by serine proteases like thrombin, a novel N-terminus is revealed on the receptor, serving as a tethered ligand that folds back into the receptor to trigger intramolecular activation (Coughlin, 2005; Macfarlane et al., 2001). This atypical process lends itself to use the amino acid sequence of a tethered ligand as a template to develop receptor-specific agonists with optimized affinity (Lerner et al., 1996). Amongst the four receptors which represent the PAR family, F2R serves as the primary thrombin receptor, and its tethered ligand sequence has been extensively elucidated (Blackhart et al., 1996; Lerner et al., 1996). The peptide sequence TFLLR-NH_2_, derived from the thrombin-cleaved N-terminus of F2R (SFLLR), has been utilized as a synthetic agonist for functional studies (Blackhart et al., 1996). It demonstrates significant selectivity for F2R, with minimal activation of other members of the PAR family. This selectivity has been validated in numerous investigations, rendering it an appropriate ligand for receptor-specific targeting in therapeutic applications (Han and Nieman, 2020). Therefore, in this study, TFLLR-NH_2_ was utilized as a targeting ligand to leverage the overexpression of F2R in ovarian cancer cells, with the objective of improving receptor-specific drug delivery via peptide-decorated LNPs.

Here we investigated F2R as a target for drug-loaded LNPs, using the TFLLR-NH_2_ peptide agonist for direct binding to the receptor on the surface of ovarian cancer cells. The LNP-conjugated peptide agonist bound to the extracellular domain of the F2R, facilitated receptor-mediated uptake of drug-loaded LNPs and increased intracellular drug accumulation in F2R-expressing ovarian cancer cells. Importantly, F2R-targeted, DOX loaded LNPs exhibited superior cellular uptake and cytotoxicity compared to non-targeted counterparts or free DOX, substantiating the potential for targeted chemotherapeutic administration.

## 2 Materials and methods

### 2.1 Materials

F2R agonist peptides with linker (GSGSGSC) and dibenzocyclooctyne (DBCO) (TFLLR-NH_2_, TFLLRGSGSGSC and TFLLR (LYS(DBCO)) were synthesized from GenScript Biotech (Kallang, Singapore). Janelia Fluor® 549, Azide was purchased from Hello Bio (Bristol, UK). Cholesterol (ovine), L-α-phosphatidylcholine, hydrogenated (Soy) (HSPC), and 1,2-distearoyl-sn-glycero-3-phosphoethanolamine-N-[methoxy(polyethylene glycol)-2000] (ammonium salt) (DSPE-mPEG2000) were purchased from Avanti research (Alabaster, USA). DSPE-PEG-Maleimide (Molecular weight (MW) 2000) was purchased from MedChemExpress (New Jersey, USA). Acetonitrile, ammonium sulphate, ethyl alcohol (pure), Float-A-Lyzers (Spectra-Por; molecular weight cut-off (MWCO) = 100 kDa), formic acid, methanol, phosphate buffered saline (PBS) powder, thin layer chromatography (TLC) aluminum sheets (silica gel 60 F_254_), Amicon® ultra centrifugal filter (molecular weight cut-off (MWCO) = 100 kDa), and sterile 0.45 µm Millex-GP syringe filter units were purchased from Sigma-Aldrich (Castle Hill, Australia). Doxorubicin hydrochloride (>98% purity) was supplied by Thermo Fisher Scientific (Scoresby, Australia). Chloroform and dimethyl sulfoxide (DMSO) were purchased from ChemSupply (Gillman, Australia). Folded capillary zeta cells (DTS1070) were supplied by Malvern Instruments Ltd (Worcestershire, UK). Milli-Q water was obtained from a Sartorius Arium® Pro Ultrapure Water System UV-T-TOC (DEU) with a resistivity of ≥18.2 MΩ⋅cm.

The ovarian cancer cell line ES-2 (RRID: CVCL_3509), Roswell Park Memorial Institute (RPMI 1640), fetal bovine serum (FBS), penicillin-streptomycin (10,000 U/mL), Dulbecco’s phosphate buffered saline (DPBS), trypsin ethylenediaminetetraacetic acid (EDTA), trypan blue solution, 3-(4,5-dimethylthiazol-2-yl)-2,5-diphenyltetrazolium bromide (MTT), bovine serum albumin (BSA), Corning® 96 and 24 well tissue culture treated microplates were purchased from Sigma-Aldrich (Castle Hill, Australia). Cell culture flasks were purchased from Greiner Bio-One (Frickenhausen, Germany). F2R siRNA was synthesized by GenePharma (Suzhou, China). Opti-MEM^TM^, lipofectamine™ RNAiMAX transfection reagent, Hoechst stain (33,342) and ProLong™ gold antifade mountant were purchased from Thermo Fisher Scientific (Scoresby, Australia). Coverslips (#1.5H 13 mm round) were purchased from Southern Cross Science (Edwardstown, Australia). Formaldehyde (32%) ampoules were purchased from Emgrid (Pooraka, Australia). Primary polyclonal F2R antibodies (26366-1-AP) were purchased from United Bioresearch (Glenorie, Australia). Secondary fluorescein isothiocyanate (FITC) labelled goat anti-rabbit IgG (H + L) antibodies were purchased from Abclonal via Genesearch. Cytofix/Cytoperm kits were purchased from BD Biosciences (San Jose, USA).

### 2.2 Conjugation of peptide and characterization

For the conjugation of TFLLR (LYS(DBCO)) with Janelia Flour dye, 10 mM stock solutions were prepared in DMSO, which were then mixed at a ratio of 1.2:1 and heated at 37 °C for 10 min. Similarly, 10 mM stock solutions of TFLLRGSGSGSC and DSPE-PEG-Maleimide were prepared in ultrapure water and ethanol, respectively. Equal volumes were mixed and heated for 1 h at 37 °C.

TLC and mass spectrometry analysis were used to confirm the intended conjugation reactions. TFLLR (LYS(DBCO)), TFLLRGSGSGSC, Janelia Fluor, DSPE-PEG-Maleimide and both final conjugated products were spotted on a TLC plate. The spotted TLC plate was placed in a TLC chamber with mobile phase prepared using chloroform, methanol and water in 8:2:0.2 (v/v) ratio. The TLC plate was stained using iodine vapor.

The conjugation of TFLLR (LYS(DBCO)) with Janelia Fluor dye was also confirmed using a TripleTOF 5,600 system that was equipped with an electrospray ionization source in positive ion mode. One µL of the conjugate was diluted in 50 µL of acetonitrile and directly infused at a flow rate of 1 μL/min. The data acquisition was performed in MS1 mode (full scan) with a scan range of m/z 300–2000. The data was analyzed using analyst software, and the theoretical mass of the conjugate was compared to the observed m/z values.

### 2.3 Design of lipid nanoparticles (LNPs) and DOX encapsulation

HSPC, cholesterol, and DSPE-mPEG2000 were combined using a mass ratio of 3:1:1. The calculated amounts for each lipid were weighed and then dissolved in ethyl alcohol (≥99.8% v/v). The solution was sonicated to complete the dissolution of lipid in ethanol and to prepare the lipid phase with the total concentration of 4 mg/mL. For peptide-conjugated LNPs, HSPC, cholesterol, DSPE-mPEG2000 and DSPE-PEG-Peptide were combined in a mass ratio of 3:1:0.5:0.5. All LNPs were produced using the microfluidics device NanoAssemblr® benchtop (Precision NanoSystems Inc., Vancouver, Canada).

In brief, the lipid stock solutions were injected into the organic inlet of the microfluidic chip (NIT0004 NanoAssemblr® cartridge). A 250 mM (pH5.5) ammonium sulphate solution was administered via the aqueous inlet to allow remote DOX loading into the formed LNPs. A total flow rate of 2 mL/min and 1:4 ratio of organic to aqueous phase was used to synthesize the LNPs. The herringbone structures of the microfluidic cartridge facilitated the mixing process. The selected total flow rate (2 mL/min) and 1:4 organic to aqueous flow ratio were based on preliminary optimization to yield blank LNPs with hydrodynamic diameters close to 100 nm and low polydispersity index (PDI) values. These conditions consistently produced stable LNPs across multiple batches and were therefore used for all formulations. The collected LNPs underwent buffer exchange using a Float-A-Lyzer® (100 kDa MWCO) dialysis chamber in PBS (pH 7.4) to allow the removal of free ammonium sulphate. The LNPs were dialyzed for up to 6 h with 50x buffer while stirring (∼250 rpm) at room temperature, with buffer replacement every 2 h. Following dialysis, a 1 mg/mL DOX solution in ultrapure water was added to the LNPs at a drug-to-lipid ratio of 1:10 (w/w). The LNPs were incubated with DOX at 60 °C for 2 h with moderate agitation to facilitate remote loading of the drug. The resulting drug-loaded LNPs with and without the targeting peptide were stored at 4 °C until further characterization.

### 2.4 Encapsulation efficiency and physical characterization

200 µL of the drug loaded LNPs were added to Amicon® ultra centrifugal filters (100 kDa MWCO). The tubes were centrifuged at 5,000 rpm for 10 min (Eppendorf centrifuge 5,424, Hamburg, Germany) and the DOX concentration in the filtrate was determined by absorption at 480 nm using a multiplate plate reader (VICTOR Nivo™). The filtrate drug concentration was compared to the total drug loading to determine the encapsulation efficiency in percent (EE%).

All four prepared LNPs (blank LNP, blank LNP-Peptide, LNP-DOX and LNP-Peptide-DOX) were further diluted in ultrapure water to determine the particle size and zeta potential at 25 °C via dynamic light scattering (DLS) using a Zetasizer Advance Ultra reader (Malvern, Worcestershire, UK). All samples were prepared in triplicate and the data acquisition and processing was performed using the built-in ZS XPLORER software (v4.0.0.683).

### 2.5 *In vitro* drug release profile

The *in vitro* release of DOX from LNPs with and without targeting peptide was investigated using a dialysis assay. Prepared LNP or pure drug solutions (1 mL) were added into Float-A-Lyzers (100 kDa MWCO) equipped with stirrer bars and positioned in plastic vials containing PBS (10 mM, pH 7.4, 10 mL) as the receiving solutions, which also included stirrer bars. The release study was performed with continuous stirring at 130 rpm at 37 °C, to mimic biological conditions. The entire receiving solution (10 mL) was collected at 1, 3, 6, 12, 24, 48 and 96 h, and substituted with fresh PBS. Finally, the concentration of DOX in the release samples from each time point were determined using a multimode plate reader (VICTOR Nivo™) measuring absorption at 480 nm, against a DOX calibration curve generated in PBS (pH 7.4). All experiments were performed in triplicate.

### 2.6 Cell culture

The Mycoalert detection kit (Lonza, Norwest, Australia) was used to confirm that all cell cultures were *mycoplasma* negative. ES-2 cells were authenticated (with 100% match) on 12th April 2021 using short tandem repeat (STR) analysis, using the Promega GenePrint® 10 system (Griffith University DNA sequencing facility, Australia). The ES-2 cell line was cultured in RPMI medium supplemented with 1% (v/v) penicillin-streptomycin and 10% (v/v) FBS, maintaining cells between passage eight and 15. Cells were maintained at 37 °C and 5% CO_2_ in an incubator until the cells showed 90% confluency.

### 2.7 Peptide binding and receptor internalization

To study the binding of TFLLR-NH_2_ to the F2R receptor expressed on ES-2 cells, cells were gently scrapped and 1 × 10^5^ cells in 100 µL PBS were added to each well in a U-bottom 96-well plate. Cells were treated with 3.9, 7.8 and 19.5 µM of peptide, which corresponded to 1×, 2× and 5× of the half maximal effective concentration (Hollenberg et al., 1997), followed by 15 min incubation at 37 °C. The incubated cells were washed twice with PBS and then stained with F2R primary antibody (1:100) for 30 min at 4 °C for cell surface staining. Cells were then stained with secondary antibody (1:100) along with live/dead stain (1:3,000) in PBS for 20 min at 4 °C. Cells were ultimately resuspended in fluorescence-activated cell sorting (FACS) buffer (PBS +2% FBS) for the flow cytometric analysis.

Similarly, the binding of TFLLR-Janelia Flour conjugate was confirmed by incubating the cells with 3.9 and 7.8 µM solutions (100 µL) for 15 min at 37 °C, followed by PBS wash. Cells were fixed using a Cytofix/Cytoperm kit according to the manufacturer’s instructions. F2R siRNA knockdown was performed as previously described (Khetan et al., 2024), to confirm the specific binding of TFLLR-Janelia Flour. The data were collected using a FACSAria™ Fusion flow cytometer (BD Biosciences, San Jose, USA) and analyzed with FlowJo™ software (Version 10, BD Biosciences, San Jose, USA).

The binding of TFLLR-Janelia Fluor was also confirmed using confocal microscopy. ES-2 cells were seeded (4 × 10^3^ cells) on coverslips for 24 h, followed by incubation with 7.9 µM (100 µL) of the conjugate for 15 min at 37 °C. Cells were washed with PBS thrice and further fixed using a solution containing 4% paraformaldehyde and 4% sucrose in PBS (400 µL) for 10 min. Cells were washed again followed by Hoechst staining (1:1,000 in PBS with 5% BSA) for 20 min at room temperature with gentle agitation. Finally, the coverslips were mounted using ProLong™ gold antifade mountant.

A Nikon A1+ confocal microscope (Nikon, Tokyo, Japan) equipped with a LU-N4/LU-N4S 4-laser unit (403, 488, 561, and 638 nm) and a Plan Apo λ ×60 oil-immersion objective lens (1.4 N.A.) at a 1.2 AU pinhole was utilized for fixed-cell confocal fluorescence microscopy. A Galvano scanner and a piezo z-stage was employed to acquire images at 512-pixel resolution with 2× line averaging, 1× zoom (0.42 μm/px), and 0.4 μm z-steps. Confocal images were processed and analyzed using the NIS Elements software (version 4.5, Nikon).

### 2.8 Cytotoxicity study

The cellular viability following exposure to blank LNP, blank LNP-Peptide, DOX only, LNP-DOX and LNP-Peptide-DOX was evaluated in ES-2 ovarian cancer cells via MTT assay. A 96-well plate was seeded with 4 × 10^3^ cells per well and incubated for 24 h at 37 °C in 5% CO_2_. After incubation, the cells were treated with 100 µL of all five groups, each dispersed in RPMI without FBS, at drug and lipid concentrations of 86 μM and 0.5 mg/mL, respectively, followed by eight serial 1:3 dilutions. Cells exposed to nanoparticle-free medium served as positive controls (100% cell viability), whereas treatment with 1% Triton X-100 was included as negative controls (0% cell viability). After 24 h of incubation, the cells were rinsed with PBS and subsequently incubated with 100 µL of 10% MTT solution in media (5 mg/mL of stock solution) for an additional 4 h. 150 μL of DMSO was introduced to the wells to solubilize the MTT formazan utilizing an orbital shaker (PerkinElmer, 100 rpm) for 10 min. Absorbance was measured at 540 nm. The absorbance data of treated cells were converted into relative cell viabilities (%) compared to positive and negative controls. Dose-response curves were generated to determine IC_50_ values using GraphPad Prism 10.

To further assess the time-dependent cytotoxicity of the formulations, MTT tests were performed at two specific concentrations (86 μM and 3.19 µM of DOX). Cells were subjected to liposomal formulations for durations of 2, 4, or 24 h, following removal of unattached particles using PBS for drug washout. Cells in the 2 and 4 h treatment groups were subsequently incubated in fresh media for an additional 22 and 20 h, respectively, to fulfil a standardized 24 h test period. Results were again represented as cell viability (%).

### 2.9 Cellular uptake of LNPs

#### 2.9.1 Live cell imaging

Live cell imaging was performed using a Nikon A1+ confocal microscope (Nikon, Tokyo, Japan) as previously described (Nturubika et al., 2024). Briefly, ES-2 ovarian cancer cells were treated with the DOX only, or the LNP-DOX and LNP-Peptide-DOX formulations at a drug concentration of approximately 86 μM and further incubated for 10 and 30 min using the Uno-combined-controller CO_2_ microscope electric top-stage incubation system (Okolab, Pozzuoli, NA, Italy). At each time point, imaging was performed using a resonant scanner at a resolution of 512 pixels (0.41 μm/pixel). A piezo z-stage was employed to acquire 18 z-steps at 0.4 μm intervals, generating 3D image stacks. A total of 100 3D frames were captured per field, with each acquisition lasting approximately 2.5 min.

#### 2.9.2 Flow cytometry

ES-2 cells were scrapped gently using the cell scrapper and 1 × 10^5^ cells/well were added as a cell suspension in a U-bottom 96 well plate. Each suspension culture was treated either with blank LNP, blank LNP-Peptide, DOX only, LNP-DOX or LNP-Peptide-DOX at a drug concentration of approximately 86 μM, along with live/dead (1:3,000) staining. The treated cells were incubated for 1 h at 37 °C, followed by PBS wash. Cells were then fixed using cytofix reagent for 20 min at 4 °C. Cells were again washed using PBS and finally suspended in FACS buffer to run the samples in the FACSAria™ Fusion flow cytometer (BD Biosciences, USA).

## 3 Results

### 3.1 Generating a TFLLR-based fluorescent probe

**FIGURE 1:**
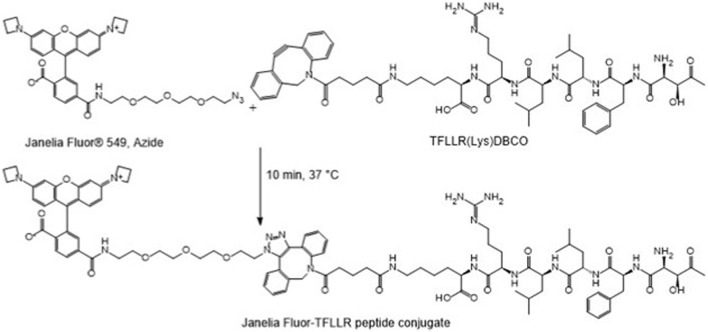
Schematic representation of the reaction between the cyclooctyne functionalized peptide (TFLLR (LYS(DBCO)) and Janelia Fluor dye, via strain-promoted azide-alkyne cycloaddition (SPAAC) click chemistry for 10 min at 37 °C.

To assess the targeting capability of the F2R agonist peptide, we used a cyclooctyne functionalized version of the peptide (TFLLR (LYS(DBCO)) and conjugated it to the fluorescent Janelia Fluor dye via strain-promoted azide-alkyne cycloaddition (SPAAC) click chemistry (Figure 1), wherein the azide moiety from the Janelia Fluor^®^ 549 molecule was linked to the cyclooctyne-modified peptide (Dommerholt et al., 2016; Sletten and Bertozzi, 2008). SPAAC provides a catalyst-free method that occurs swiftly under physiological conditions. The selective reaction occurs without disrupting natural biological processes or cellular components, hence reducing the likelihood of off-target chemical interactions or cytotoxicity in subsequent applications (Dommerholt et al., 2016; Sletten and Bertozzi, 2008).

**FIGURE 2:**
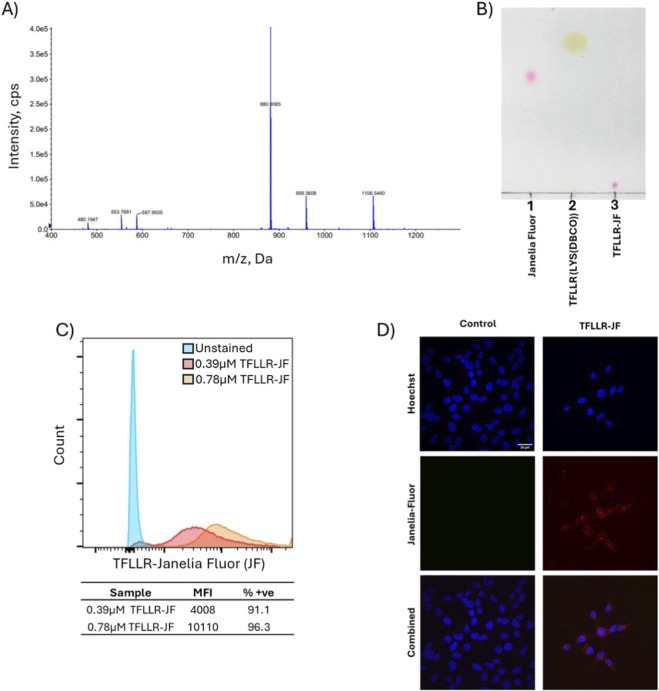
Characterization of TFLLR-Janelia Fluor conjugate and binding of the conjugate to the F2R receptor on ES-2 cells. **(A)** Analysis of the (TFLLR (LYS(DBCO))-Janelia Fluor (TFLLR-JF) conjugate via TripleTOF mass spectrometry. The peaks at m/z 553.8 and 880.9correspond to doubly charged ions ([M+2H]^2+^) of the pure peptide and the anticipated conjugate, respectively. The peak at m/z 1,106.5 represents [M + H]^+^ ions of parent peptide. **(B)** Thin-layer chromatography (TLC) of the conjugation product. Lane 1: Janelia Fluor; Lane 2: unmodified TFLLR (LYS(DBCO)) peptide; Lane 3: TFLLR-JF conjugate. TLC was run in a mobile phase with chloroform, methanol, and water at a ratio of 8:2:0.2 (v/v) and stained using iodine vapor. **(C)** ES-2 cells were incubated with 0.39 and 0.78 μM of the TFLLR-JF conjugate corresponding to 1× and 2× concentrations relative to the published EC_50_ value of 0.39 μM (Hollenberg et al., 1997). Histograms are shown for unstained, 0.39 and 0.78 μM TFLLR-JF conjugate, as indicated in the figure legend. Median fluorescent intensities (MFI) and percentage positive cells (% +ve) were obtained for each sample. **(D)** Confocal microscope images depicting TFLLR-JF binding to ES-2 ovarian cancer cells. ES-2 cells were treated with media only (left panel) and 0.78 μM concentration of TFLLR-JF (right panel). The top and middle images show Hoechst stain (blue) and Janelia Fluor (red). Composite images are given at the bottom. The scale bar is 20 μm.

The reaction was carried out as described in the methods part using a slight molar excess of peptide relative to the dye. We performed TripleTOF 5,600 mass spectrometry to verify effective conjugation between the cyclooctyne-functionalized peptide (exact mass 1,105.6; MW 1106.3 Da) and the azide-functionalized Janelia Fluor dye (exact mass 654.3; MW 654.7 Da), (Figure 2A). The anticipated exact mass and MW of the final triazole-linked conjugate was 1759.9 and 1761.1 Da. In the spectra, two prominent signals were observed at m/z 1,106.5 and m/z 880.9 (Figure 2A), which corresponded to the [M + H]^+^ ions of parent peptide and doubly charged [M+2H]^2+^ ions of the complete conjugate, respectively. A low-intensity signal at m/z 553.8 was also observed for doubly charged [M+2H]^2+^ ions for the parent peptide. TripleTOF predominantly produces singly charged ions; nevertheless, doubly charged species may emerge due to the terminal amine and arginine group in the peptide which could both be protonated. The presence of accurate signals for the reactant and product confirmed the effective conjugation via SPAAC.

Furthermore, the efficacy of the cyclooctyne and Janelia Fluor dye SPAAC conjugation reaction was qualitatively assessed via TLC (Figure 2B). The results confirmed the free reactants (lanes one and 2) and the fluorophore-conjugated peptide (lane 3). The free fluorophore (lane 1) and the DBCO functionalized peptide (lane 2) produced a fast-migrating distinct single spots on the iodine developed chromatogram. Notably, lane 3 (conjugate) exhibited a solitary pink spot at the base of the TLC plate, which was absent in both control lanes. This newly identified, lower-migrating species indicated heightened polarity and effective peptide-fluorophore conjugation using SPAAC. The pink hue of the conjugate confirmed that the fluorophore retained its chemical integrity post-reaction. Collectively, these data technically validated the successful generation of a Janelia Fluor conjugated TFLLR peptide, which has been referred to as TFLLR-JF throughout the remaining text.

We have previously evaluated F2R expression in five different ovarian cancer cell lines (Khetan et al., 2024). The ES-2 cell line showed very high F2R gene and cell surface protein expression and was therefore selected as the preferred *in vitro* model for this study. To directly assess receptor engagement, we treated cells with TFLLR-JF and monitored fluorescence intensities by flow cytometry (Figure 2C). The fluorescent peaks appeared to be broad, possibly indicating partial peptide dissociation during handling, which is prevalent in flow cytometry studies to assess ligand-receptor interactions (Owensby et al., 1989; Sorkin and von Zastrow, 2009). This could also reflect both intrinsic variability in F2R expression across ES-2 cells and differences in membrane presentation of the peptide–cyclooctyne construct, which lacks a linker and may adopt variable orientations. Cells treated with 0.39 and 0.78 µM of the conjugate for 15 min demonstrated a clear dose-dependent increase in median fluorescence intensity (MFI). These dynamics were consistent with the assumption that the dye-labelled TFLLR-JF peptide was biologically active and could specifically engage its receptor, driving strictly regulated internalization and recycling (Grimsey et al., 2016). To further consolidate these assumptions, confocal fluorescence microscopy was used to visually verify cellular binding and uptake of the TFLLR-JF conjugate with ES-2 cells. The cells were incubated for 15 min at 37 °C with either (i) tissue culture media only (Figure 2D, left panels) or (ii) 0.78 μM of the TFLLR-JF conjugate prepared in media (Figure 2D, right panels). The cells were fixed with 4% paraformaldehyde, washed with PBS, stained with Hoechst, then imaged using fixed exposure times. The control group (left panels) exhibited pronounced nuclear Hoechst staining with no background signal in the absence TFLLR-JF (Figure 2D). Conversely, the treated cells (right panel) displayed a substantial fluorescent signal (red) which appeared to be localized at or in close proximity to the cell membrane and within the cytoplasm, affirming specific binding of the TFLLR-JF conjugate and suggesting potential early-stage internalization. The brief incubation period (15 min) indicated that the compound rapidly bound to the target receptor on ES-2 cells, with partial internalization observed, followed by peripheral and intracellular localization. The observed pattern was consistent with the flow cytometry results, and implied that the F2R targeting peptide was successfully bound to the receptor and was internalized into ovarian cancer cells. The cell surface binding of the TFLLR-JF shown via flow cytometry and confocal imaging suggested that this ligand could be suitable for targeted ovarian cancer delivery applications.

### 3.2 Testing TFLLR as a suitable F2R ligand

**FIGURE 3:**
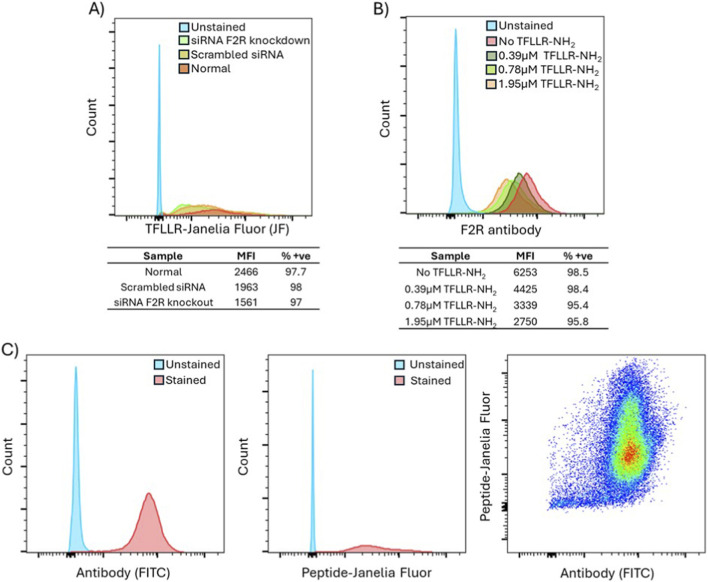
**(A)** TFLLR-JF binding analysis on ES-2 ovarian cancer cells: The F2R was knocked down using specific siRNA, and TFLLR-JF binding was studied at 0.78 μM, which corresponded to 2× EC_50_ (Hollenberg et al., 1997). The histograms indicate the amount of TFLLR-JF conjugate binding to unstained, siRNA F2R knockdown, scrambled siRNA control, and normal cells, respectively. **(B)** Cells treated with unlabeled peptide (TFLLR-NH_2_) at 1×, 2× and 5× EC_50_, subsequently fixed and stained with a receptor-specific antibody. The histograms correspond to unstained, no TFLLR-NH_2_, or 0.39, 0.78, and 1.95 μM TFLLR-NH_2_. **(C)** Surface co-binding of F2R antibody and TFLLR-JF peptide on ES-2 ovarian cancer cells. Binding of F2R primary antibody on ES-2 cells (left panel), visualised with FITC-labelled secondary antibody, binding of peptide-Janelia Fluor conjugate (middle panel), and binding of both antibody and conjugate to ES-2 cells (right panel). The histogram indicates unstained and stained samples respectively.

To validate the specificity and functionality of the TFLLR peptide binding, two sets of experiments were performed: 1) the F2R receptor expression was knocked down using siRNA followed by the binding of TFLLR-JF (Figure 3A) and 2) receptor internalization was triggered with the TFLLR-NH_2_ agonist proving reduced cell surface receptor density using a specific F2R antibody (Figure 3B). Flow cytometry analysis demonstrated that the cells subjected to normal conditions (without knockdown) exhibited the highest binding to TFLLR-JF, as evidenced by a fluorescence shift to the right, compared to the unstained control (Figure 3A). Conversely, cells exposed to siRNA F2R knockdown exhibited a significant reduction in TFLLR-JF binding, which was consistent with a decrease in receptor availability on the cell surface. Cells treated with scrambled siRNA demonstrated only a slight decrease, likely due to non-specific effects of the transfection procedure. In conclusion, F2R knockdown decreased receptor availability, resulting in reduced TFLLR-JF binding. This also supported the hypothesis that the signal observed in the previous data (Figure 2C) was receptor-mediated, rather than the result of non-specific or off-target cell surface interactions.

To further demonstrate the specificity of TFLLR binding, increasing doses of TFLLR-NH_2_ were utilized (Figure 3B). Cells were exposed to 0.39, 0.78 and 1.95 µM of TFLLR-NH_2_ (1×, 2× and 5× EC_50_) for 15 min at 37 °C followed by labelling with an anti-F2R antibody on ice. This led to a dose-dependent reduction in the antibody-detected surface signal (MFI) in flow cytometry experiments, aligning with agonist-induced receptor internalization. This decrease indicated the internalization of F2R subsequent to activation by TFLLR-NH_2_. Nonetheless, it could be possible that the reduced antibody signal may potentially result from competition between the antibody and ligand for the identical binding site. To address this possibility, we conducted control studies (Figure 3C), which verified that the antibody and TFLLR-NH_2_ interacted with separate binding sites, thereby ruling out an artifact due to competition.

Overall, our results corroborate previously published data identifying the TFLLR-NH_2_ peptide as a functional receptor agonist and we further demonstrated it effectively triggers surface receptor internalization in a specific and concentration-dependent manner. These findings strengthened our rationalization for the design of a drug carrier functionalized with the TFLLR-NH_2_ ligand to target F2R over-expressing ovarian cancer cells.

### 3.3 Design and characterization of F2R-targeted lipid nanoparticles

**FIGURE 4:**
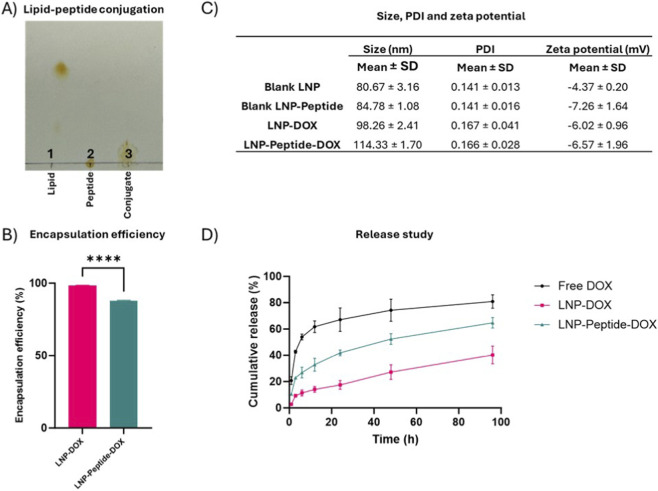
Design and characterization of peptide-decorated LNPs. **(A)** Confirmation of lipid-peptide conjugation using TLC. Lanes 1, two and three represent pure lipid (DSPE-PEG-maleimide), pure peptide ligand (TFLLR-GSGSGSC) and the conjugated product, respectively. TLC was run in a mobile phase with chloroform, methanol, and water in 8:2:0.2 (v/v) ratios and was stained using iodine vapor. **(B)** Encapsulation efficiency percentage (%) for LNPs loaded with the chemotherapeutic drug DOX. The encapsulation efficiency for LNP-DOX (pink) and LNP-Peptide-DOX (green) was 97% and 88%, respectively. Statistical analysis included Welch’s test, **** = p < 0.0001. **(C)** Hydrodynamic diameter, PDI and zeta potential for blank LNP, blank LNP-Peptide, LNP-DOX and LNP-Peptide-DOX. **(D)** *In vitro* release study for LNP-DOX (pink) and LNP-Peptide-DOX (green) over 96 h using a dialysis membrane assay and quantified by reading the DOX absorbance at 480 nm. Free DOX control is given in black. Cumulative release (%) over time is shown for all samples. Data presented as mean ± SD.

A F2R-targeted LNP was designed using a HSPC, cholesterol, and DSPE-PEG based composition (Alyane et al., 2016; Tejada-Berges et al., 2002). To conjugate the TFLLR-NH_2_ ligand to DSPE-PEG-maleimide, we designed an extended peptide (TFLLRGSGSGSC) with a flexible and polar GSGSGS linker, and a C-terminal cysteine (C) residue for coupling via click chemistry. The successful conjugation of lipid and peptide was technically validated using TLC, revealing a unique migration characteristic for the conjugate in contrast to the free compounds (Figure 4A). The migration behavior on the TLC indicated the effective covalent bonding by thiol-maleimide chemistry, is a commonly employed method for targeted ligand attachment to lipid anchors (Fontaine et al., 2015). This procedure was crucial for guaranteeing robust peptide presentation on the LNP surface following lipid particle assembly.

LNPs were synthesized utilizing a microfluidic mixing system, and DOX was encapsulated into the LNPs using a remote loading approach that was based on an established ammonium sulphate gradient procedure (Alyane et al., 2016). LNPs were incubated with DOX for 2 h at 60 °C followed by buffer exchange to PBS, which enabled active drug loading driven by transmembrane ion gradients. This technique facilitates the effective encapsulation of amphiphilic weak bases by utilizing a transmembrane pH gradient, leading to drug precipitation within the liposomal core and achieving high EE% (∼90%) with minimum premature leakage (Alyane et al., 2016). The EE% of non-targeted LNPs surpassed 95%, whilst the targeted, peptide-decorated LNPs maintained a substantial EE% of approximately 88% (Figure 4B). The small decrease in EE% observed in the targeted formulation can be explained by minor steric modifications in the lipid bilayer packing caused by the surface-bound ligands (Pande, 2024). Importantly, both formulations achieved the standard EE% limits necessary for therapeutic efficacy, typically ranging between 70% and 90% (Banerjee and Sen, 2024).

LNP characterization by DLS revealed a mean hydrodynamic diameter of 98.26 nm for LNP-DOX, and 114.33 nm following the peptide decoration on the surface (Figure 4C). All formulations had polydispersity index (PDI) values below 0.2, signifying a narrow-dispersed population (Danaei et al., 2018). The zeta potential of all LNPs remained approximately neutral, as expected, due to the presence of PEG on the surface, which has no surface charge. These dimensions and surface characteristics were within the ideal ranges for optimal EPR effects, facilitating intra tumoral accumulation and promoting active receptor-mediated uptake (Rommasi and Esfandiari, 2021).

The *in vitro* release of DOX was assessed in PBS, pH 7.4 at 37 °C via a dialysis membrane assay. The 96 h *in vitro* release assay represents the physicochemical stability and long-term behavior of the formulation under cell-free conditions. Importantly, only approximately 10% of DOX was released during the first hour. In combination with rapid cellular uptake, it strongly suggests that most of the drug will remain encapsulated. Hence, only negligible cytotoxic effects can be caused by freely diffusing compound. Free DOX demonstrated fast diffusion, with around 65% release occurring within 24 h (Figure 4D). Conversely, both targeted and non-targeted LNPs exhibited prolonged drug release, with approximately 15%–25% of drug released in an equivalent timeframe (Figure 4D). The peptide-functionalized LNPs exhibited somewhat faster release rates compared to LNP-DOX, presumably due to enhanced bilayer and modified lipid packing density resulting from the conjugated ligand. Although both targeted and non-targeted LNPs showed partial drug release in PBS within the first 24 h, this *in vitro* condition does not reflect the intracellular environment where DOX is released following receptor-mediated endocytosis. Once internalized, the nanoparticles are trafficked into early and late endosomes, where progressive acidification (pH 5.0–6.0) promotes destabilization of lipid membranes and accelerates DOX diffusion into the cytoplasm and nucleus. Collectively, these observations technically validate the effective design of an LNP pertinent for the further development of a therapeutic system that can include F2R-targeting peptides, without compromising drug loading or encapsulation efficiency. The formulation preserved essential physicochemical features akin to FDA-approved liposomal systems such as Doxil^®^, while incorporating receptor selectivity via strategic ligand modification.

Subsequently, we aimed to ascertain that surface functionalization with an F2R-targeting peptide augments cellular internalization and cytotoxic activity in ovarian cancer cells. To address this, we compared targeted and non-targeted LNPs employing flow cytometry, live imaging confocal microscopy and the MTT cell viability assay, to evaluate absorption and functional effects.

### 3.4 Cellular uptake of targeted and non-targeted LNPs

**FIGURE 5:**
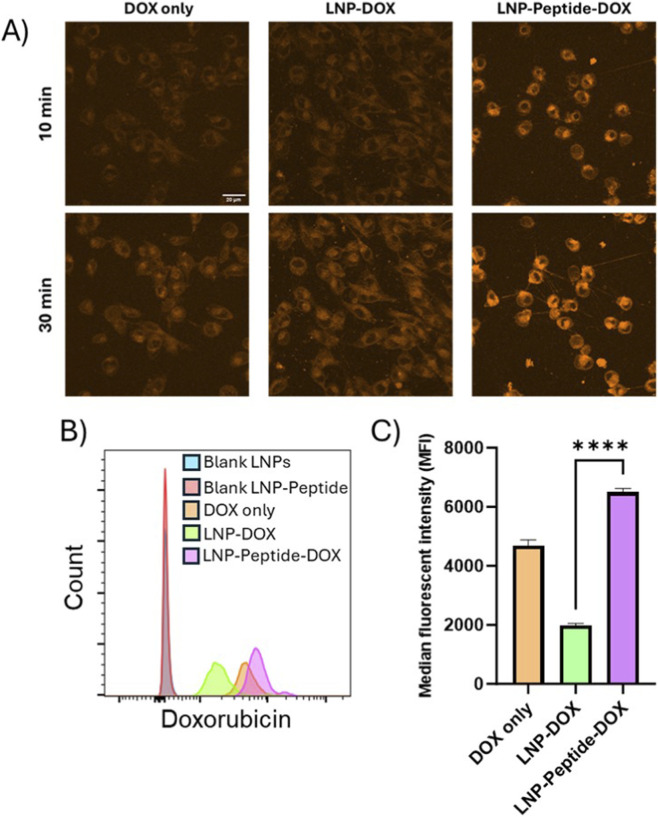
Cellular uptake of targeted and non-targeted LNPs in ES-2 cells. **(A)** Confocal images of 10 min (top panels) and 30 min (bottom panels) incubation time point for DOX only, LNP-DOX and LNP-Peptide-DOX (scale bar = 20 µm). **(B)** Flow cytometry analysis for the uptake of blank LNPs, blank LNP-Peptide-DOX, DOX only, LNP-DOX and LNP-Peptide-DOX after 1 h incubation at 37 °C. **(C)** Median fluorescent intensity (MFI) compared for DOX only, LNP-DOX and LNP-Peptide-DOX. Statistical analysis included one-way Anova followed by Tukey’s multiple comparisons test, **** = p < 0.0001. Data presented as mean ± SD.

To first assess the cellular uptake dynamics of liposomes, we performed live-cell confocal imaging using the DOX only, LNP-DOX and LNP-Peptide-DOX formulations with approximately 86 μM drug. DOX has very robust intrinsic fluorescence properties which was used to directly visualize cellular absorption and distribution (Shah et al., 2017; Uyar et al., 2020) (Figure 5A). At the 10 min interval, cells subjected to LNP-Peptide-DOX exhibited strong intracellular fluorescence, signifying rapid binding/internalization. In contrast free DOX and LNP-DOX controls produced weaker and more diffuse signals, suggesting a more non-specific distribution indicative of slower and passive uptake. After 30 min, the LNP-Peptide-DOX formulation demonstrated increased accumulation with evidence of labelling at or near the cell surface in punctate structures consistent with endosomal vesicles and nanotubes/structures connecting between the ES-2 ovarian cancer cells. Some of the labelling appeared to be in cap-like structures, suggesting a possible intermediate in the receptor mediated uptake process. The absorption of untargeted LNP-DOX and free DOX remained limited and comparable to the earlier 10-min time point.

We next increased the incubation time to 1 h with the same LNP drug concentrations and utilized flow cytometry to quantitatively evaluate cellular fluorescence, comparing targeted and non-targeted formulations (Figures 5B,C). The LNP-Peptide-DOX formulation displayed an MFI per cell of approximately 6,000 after 1 h of exposure at 37 °C (Figure 5C). In comparison, cells exposed to LNP-DOX exhibited a 3× lower MFI of approximately 2000. These results demonstrate that F2R targeted LNPs interact with target cells at a substantially higher rate than non-targeted LNPs. The amplified signal further verifies that the receptor plays a functionally significant role in promoting cellular interaction and uptake. The increased MFI of approximately 4,500 for free DOX indicated increased passive uptake over extended exposure time. This further indicates that the encapsulation of DOX within LNPs inhibits the intracellular delivery, whereas the integration of receptor-specific peptides onto LNP formulations significantly improves absorption efficiency. This underscores the enhanced efficacy of targeted LNP-peptide systems, potentially providing optimized delivery methods for forthcoming *in vivo* applications.

These findings confirmed that the F2R-targeting peptide not only enhances receptor recognition but also markedly expedites the internalization of the nanoparticle carrier system. The initial uptake of LNP-Peptide-DOX within cells indicated the potential for intracellular drug release, which may enhance the cytotoxic effect of the drug. To translate these uptake dynamics into functional results, we further assessed the cytotoxic response over different exposure times. This enabled us to evaluate whether improved cellular uptake correlated with enhanced cytotoxic effects of these LNPs.

### 3.5 Assessment of LNP-Peptide-DOX cytotoxicity

**FIGURE 6:**
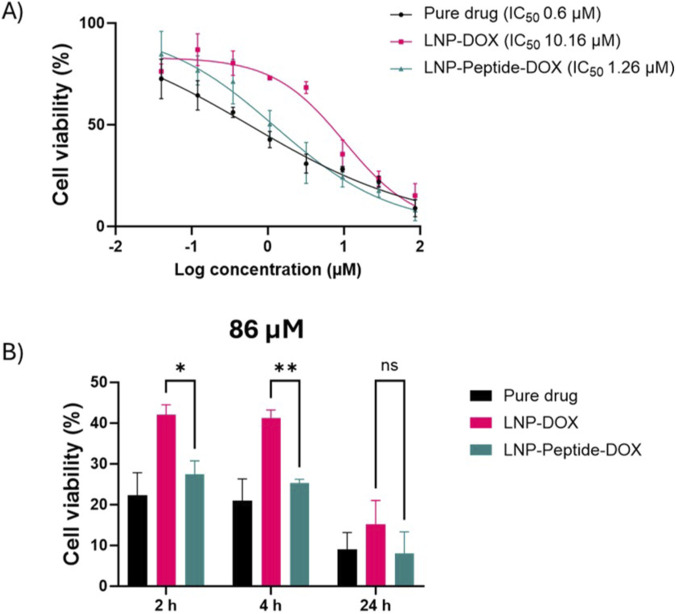
Cytotoxic effect of pure drug, LNP-DOX and LNP-Peptide-DOX on the ES-2 ovarian cancer cell line. **(A)** IC_50_ curve generated after 24 h incubation for pure drug, LNP-DOX and LNP-Peptide-DOX using eight concentrations with IC_50_ values of 0.6, 10.16 and 1.26 µM, respectively. **(B)** Cellular viability was assessed at 86 µM concentration, following 2, 4 and 24 h incubations with pure drug, LNP-DOX and LNP-Peptide-DOX. All experiments were conducted in triplicates. Statistical analysis includes Mann-Whitney test, * = p < 0.05, ** = p < 0.01, ns = non-significant. Data presented as mean ± SD.

Cell viability dose-response curves were generated using eight drug concentrations for the pure drug as a control, LNP-DOX and LNP-Peptide-DOX (Figure 6A). As expected, treatment with free DOX resulted in a significant reduction in cell viability (IC_50_ = 0.6 µM), consistent with its established mechanism of DNA intercalation, leading to indirect topoisomerase II inhibition (Nitiss, 2009). The LNP-Peptide-DOX formulation exhibited slightly weaker cytotoxicity (IC_50_ = 1.26 µM) relative to free DOX, but much stronger effects relative to LNP-DOX (IC_50_ = 10.16 µM), confirming that F2R-mediated endocytosis improves the efficacy of drug delivery into ES-2 cells.

To elucidate the time- and dose-dependent characteristics of these formulations, ES-2 cells were incubated with LNP-DOX and LNP-Peptide-DOX for 2, 4, and 24 h at a concentration of 86 µM (Figure 6B). Following the designated incubation periods, the drug-containing media were discarded, cells were rinsed with PBS, and fresh media were introduced for ongoing culture to fulfil a total assessment duration of 24 h, as outlined in the methods section. At 86 µM (Figure 6B), a notable difference was detected as early as 2 and 4 h, with LNP-Peptide-DOX exhibiting superior cytotoxicity relative to LNP-DOX. This initial effect was probably caused by fast receptor interaction and internalization, facilitating expedited intracellular distribution of DOX. By 24 h, the cytotoxicity of both formulations seemed to plateau, exhibiting no statistically significant difference, potentially attributable to receptor saturation or the attainment of maximal intracellular DOX accumulation in both groups. Although the LNP-DOX release profile showed some passive DOX leakage in the initial 2 h (Figure 4D), the increase in cellular uptake shown at 10, 30 and 60 min (Figure 5) suggested that the cytotoxic differences seen at early time points were mostly due to the specific uptake of LNP-Peptide-DOX. Despite higher early uptake, LNP-Peptide-DOX shows delayed cytotoxicity relative to free DOX due to the requirement for endosomal trafficking and escape, whereas free DOX rapidly diffuses into the nucleus and produces an earlier cytotoxic response.

To further explore these effects, we assessed a lower concentration of 3.19 µM, chosen based on the most significant differential response noted between formulations in the IC_50_ screening. At 3.19 µM sub-saturating dose (Supplementary Figure S1), no significant difference was observed between LNP-DOX and LNP-Peptide-DOX at 2 and 4 h, likely due to limited DOX concentrations at the target site. However, by 24 h, LNP-Peptide-DOX demonstrated significantly enhanced cytotoxicity (p < 0.05), presumably attributable to the time-dependent internalization and accumulation of the peptide-targeted particles. These findings indicate that the advantages of receptor targeting are more evident at sub-saturating drug doses with extended exposure, allowing active uptake pathways to fully function. This supports the idea that receptor-targeted design is functionally important for enhanced drug delivery.

Lastly, cells were also exposed to blank LNPs with and without peptide ligand, and the MTT assay revealed only negligible effects on cell viability (Supplementary Figure S2). These control experiments demonstrated that the unmodified and the peptide-conjugated lipid matrix are biocompatible and do not elicit off-target cytotoxic effects. These cytotoxicity studies highlight the time- and concentration-dependent benefits of peptide-mediated delivery. Receptor-targeted LNPs have significant advantages at lower dosages and extended incubation periods, while simultaneously displaying improved early efficacy at elevated concentrations, underscoring their potential for both prolonged and immediate therapeutic uses.

The chosen experimental time windows reflect different biological processes relevant to targeted delivery. The 30–60 min uptake experiments capture the early internalization window characteristic of GPCR-mediated endocytosis, which occurs rapidly and is driven by receptor activation. The previous results in Figure 4D demonstrated only minimal drug release from LNPs during this process, confirming that uptake is driven by nanoparticle–receptor interactions rather than passive diffusion of free DOX. In contrast, the 24 h cytotoxicity assay incorporates the cumulative effects of intracellular trafficking, nuclear delivery of DOX, and sustained release over the exposure period. Together, these complementary time frames reflect a sequential mechanism: the rapid receptor-mediated uptake followed by prolonged intracellular DOX availability, which underpins the enhanced therapeutic effect of the targeted LNPs. These findings endorse the mechanistic justification for integrating ligand-based targeting in nanoparticle design for drug delivery applications. Utilizing known receptor trafficking routes, tailored nanoparticles can surpass diffusion-limited absorption and potentially decrease the necessary therapeutic dosage of drugs by boosting the drug distribution ratio between cancer and healthy cells, thereby decreasing systemic toxicity and enhancing tumor-specific efficacy.

## 4 Discussion

The development of strategies for tumor-selective drug delivery remains a critical unmet need in therapeutic oncology, especially for chemotherapy-resistant and recurrent malignancies such as ovarian cancer. Lipid-based nanocarriers have revolutionized the pharmacokinetics and toxicity profiles of traditional chemotherapeutics; yet their clinical efficacy has been constrained by sub-optimal tumor specific accumulation and restricted cellular uptake (Mandal, 2025; Ning et al., 2024). Passive targeting techniques that are dependent on the EPR effect frequently result in inadequate drug concentrations in the tumor and tumor microenvironment (Attia et al., 2019; Li et al., 2023). Given these limitations, ligand-mediated active targeting has emerged as a more efficient strategy to deliver drug loaded nanocarriers directly into tumor cells by leveraging selective receptor recognition and endocytic uptake.

Our research focused on validating the concept of ligand-mediated targeted delivery of a chemotherapeutic drug into ovarian cancer cells. We demonstrated that a GPCR-targeting peptide agonist can be efficiently utilized to functionalize LNP-DOX, resulting in markedly enhanced cellular interaction, uptake and cytotoxicity against ovarian cancer cells. Our methodology involves a small-molecule peptide ligand conjugated to LNPs using a relatively simple and rapid click chemistry, enabling precise and scalable delivery with translational potential. This targeted LNP technology represents an early-stage drug discovery model with potential for next stage evaluation in pre-clinical small animal cancer models. Noteworthy, there are over 100 human GPCRs known to be activated by peptides, many of which are overexpressed in various malignancies, but with F2R playing an important role for ovarian cancer. Our work provides a framework for the development of a personalized therapeutic platform by combining GPCR expression profiling in different tumor biopsies with the use of customized ligands from a pre-validated peptide library. This could facilitate the modular assembly of clinically established small-molecule therapeutics, loading them into LNPs, which can then be fitted with specific targeting ligands to accelerate the translation of future receptor-guided precision nanomedicines into clinical practice.

A strength of the LNP-Peptide-DOX system evaluated here is the use of a clinically established lipid formulation, analogous to that of Doxil^®^ (HSPC:Cholesterol:DSPE-PEG), which is generally recognized as having good biocompatibility, prolonged circulation duration, and effective encapsulation of both hydrophilic and hydrophobic chemotherapeutics (Barenholz, 2012). The application of the ammonium sulphate gradient technique for active drug loading achieved elevated encapsulation efficiency and stable retention of DOX within the lipid nanoparticle core. The physicochemical investigation demonstrated that peptide conjugation did not adversely affect the size or stability of the nanoparticles, which is essential for scalable production and *in vivo* pharmacokinetics. This indicated that the LNP-Peptide-DOX technology developed and technically validated here *in vitro,* might be directly utilized in an *in vivo* pre-clinical animal model to further validate the utility for ovarian cancer treatment.

Cytotoxicity testing revealed that the enhanced targeting and uptake of DOX cargo resulted in superior cancer cell killing efficacy. Free DOX, as expected, caused extensive cell death due to its rapid diffusion into the cells, whereas the targeted LNPs appeared to elicit a more regulated cytotoxic response. Importantly, blank LNPs, with and without peptide, demonstrated no observable toxicity, confirming excellent biocompatibility of the drug delivery system. The enhanced efficacy of the LNP-Peptide-DOX technology underscores the importance of cell-specific targeting for optimal therapeutic activity and minimal off-target consequences, a vital prerequisite for the development of precision nanomedicines. Although we confirmed that the TFLLR ligand induces F2R internalization, we did not directly image receptor trafficking following LNP-Peptide-DOX treatment. Future studies using dual-labelled LNPs and receptor markers will be helpful to visualize receptor–nanoparticle co-internalization. The interesting observation of labelling in nanotube-like structures suggested that this technology may also be amenable to access inter-cellular transport mechanisms; further enhancing the penetration and dispersion of the drug within the cancer. In addition, the accelerated intracellular uptake and delivery of LNP-Peptide-DOX in comparison to Peptide-DOX substantiates the kinetic benefit afforded by active targeting.

From a translational standpoint, our study advances the developing field of receptor-guided nanoparticles by demonstrating feasibility of tailoring drug carriers to the molecular profile of the tumor (Palanisamy and Mandal, 2025). Our findings indicate that small-molecule or peptide-based ligands, when strategically designed and integrated into clinically established LNP platforms, present a significant opportunity to enhance specific drug delivery into cancer cells. Future translation into the clinic harbors the potential to provide novel monitoring technology and maintenance treatments to reduce the risk of recurrence after cytoreductive surgery, proving an alternative to conventional chemotherapy. The combination of this technology with existing therapies may also provide synergistic advantages, including enhanced safety profiles and dose reduction. Collectively, these integrative strategies have the potential to expand the therapeutic window and optimize the benefit-risk ratio for patients with advanced-stage ovarian cancer.

## 5 Limitations and future direction

The present study offers substantial evidence for the efficacy of F2R-targeted lipid nanoparticles in improving intracellular delivery and cytotoxicity of doxorubicin in ovarian cancer cells; nonetheless, several limitations warrant careful consideration. One key challenge is the inadequate drug retention within LNPs, even when utilizing the ammonium sulphate gradient loading approach. While this method exhibited satisfactory encapsulation efficacy, there was some premature drug leakage that could potentially compromise specific drug delivery into cancer cells. The drug-loading method could be further optimized by additional developmental research, including the covalent conjugation of DOX with cleavable pH-sensitive linkers (DOX-hydrazone-PEG) within the nanoparticles. This technique would not only improve the encapsulation efficiency but also facilitate controlled intracellular release after receptor-mediated endocytosis. Additional studies are needed to delineate the receptor specificity of this targeted delivery approach, including the use of alternative control cell lines with minimal or no F2R expression. Future work will also incorporate quantitative modelling of ligand density versus receptor abundance, and evaluation across cell lines with differing F2R expression, to define the sensitivity range of this targeting strategy. While the use of *in vitro* models offers substantial mechanistic insights, they fail to accurately represent the *in vivo* microenvironment of the malignancy, which may influence receptor accessibility, nanoparticle localization, peptide ligand stability and therapeutic response. Thus, an *in vivo* study is warranted as a next step in the specificity assessment and validation process. Protein adsorption on the LNP surface is another significant factor to be considered, as it can affect nanoparticle stability, biodistribution, and may obstruct ligand–receptor interactions essential for targeted delivery. Therefore, it will be critical to test targeted formulations with *in vivo* models to gain insights into biodistribution, pharmacokinetics, and antitumor activity in a physiological setting.

The flexibility of this new technology platform can easily be extended beyond F2R and DOX, including additional GPCRs and payloads. In summary, ligand-decorated LNPs provide an excellent drug delivery platform, with a well-optimized nanocarrier architecture that can be adapted for use across different tumor types. Such adaptability enables combination strategies, such as co-delivery of synergistic chemotherapeutic drugs or integration with immune modulators, to boost efficacy while also overcoming drug resistance. This nanocarrier platform, combines effective formulation of drugs with precise targeting, hence represents a potential next-generation approach to personalized drug delivery in ovarian and other solid tumors.

## Data Availability

The original contributions presented in the study are included in the article/Supplementary Material, further inquiries can be directed to the corresponding authors.
