# Supplementary material for: Targeting F2R/PAR1 with ligand decorated lipid nanocarriers for enhanced drug delivery into ovarian cancer cells
Source: Front Drug Deliv. 2026 Jan 13;5:1727958. doi: 10.3389/fddev.2025.1727958 (PMC12835398; doi:10.3389/fddev.2025.1727958)
Supplement: Supplementary file 1 [file Supplementaryfile1.docx]

**Supplementary information**

**Targeting F2R/PAR1 with lipid nanocarriers for enhanced drug delivery into ovarian cancer cells**

Riya Khetan^1^, Weranga Rajapaksha^1^, Bukuru Nturubika^2^, Todd A Gillam^c^, Doug A Brooks^2^, Sanjay Garg^1^, Anton Blencowe^1,4^, Hugo Albrecht1^,*^, Preethi Eldi^2,*^,

^1^ Centre of Pharmaceutical Innovation, UniSA Clinical and Health Sciences, University of South Australia, Adelaide, South Australia 5000, Australia

^2^ Clinical and Health Sciences, University of South Australia, Adelaide, South Australia 5000, Australia

^3^School of Mathematics, Statistics, Chemistry and Physics, Murdoch University, Murdoch, Western Australia 6150, Australia

^4^Applied Chemistry and Translational Biomaterials Group, Centre of Pharmaceutical Innovation, UniSA Clinical and Health Sciences, University of South Australia, Adelaide, South Australia 5000, Australia

* Corresponding author emails:

[preethi.eldi@unisa.edu.au](mailto:preethi.eldi@unisa.edu.au) and [hugo.albrecht@unisa.edu.au](mailto:hugo.albrecht@unisa.edu.au)


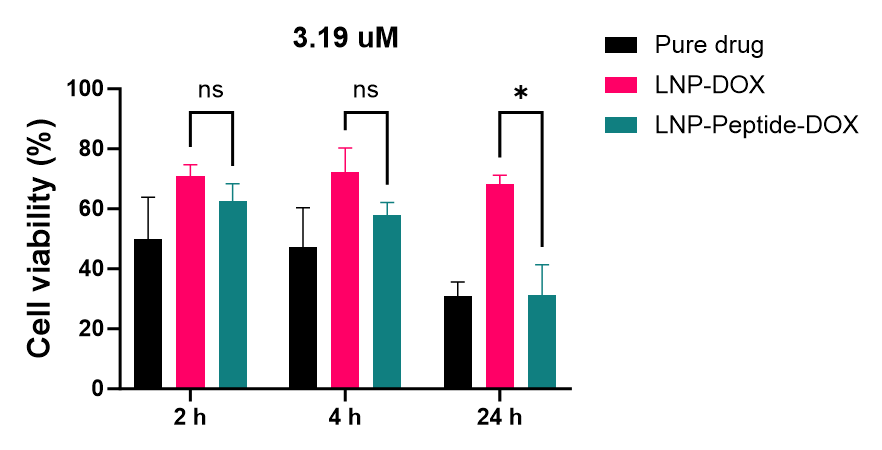


**Figure S1.** Cytotoxic effect of pure drug, LNP-DOX and LNP-Peptide-DOX on the ES-2 ovarian cancer cell line. Cellular viability tested at 3.19 µM concentration, with the incubation of 2, 4 and 24 h for pure drug, LNP-DOX and LNP-Peptide-DOX. All experiments were conducted in triplicates. Statistical analysis includes Mann-Whitney test, * = p < 0.05, ns = non-significant. Data presented as mean ± SD.

**Figure S2.** Cytotoxic effect of blank liposomes on ES-2 ovarian cancer cell line. Blank LNPs and Blank-LNP-Peptide were used to incubate for 2 h, 4 h and 24 h on ES-2 cells. All the samples resulted in more than 80% of cell viability.
